# Persisting atypical and cystic forms of *Borrelia burgdorferi *and local inflammation in Lyme neuroborreliosis

**DOI:** 10.1186/1742-2094-5-40

**Published:** 2008-09-25

**Authors:** Judith Miklossy, Sandor Kasas, Anne D Zurn, Sherman McCall, Sheng Yu, Patrick L McGeer

**Affiliations:** 1 Kinsmen Laboratory of Neurological Research, University of British Columbia, 2255 Wesbrook Mall, Vancouver, B.C. V6T1Z3, Canada; 2 Laboratoire de Physique de la Matière Vivante, Ecole Polytechnique Fédérale de Lausanne, 1015 Lausanne, Switzerland and Département de Biologie Cellulaire et de Morphologie, Université de Lausanne, 1005 Lausanne, Switzerland; 3 Department of Experimental Surgery, Lausanne University Hospital, CH-1011 Lausanne, Switzerland; 4 Pathology Laboratory, U.S. Army Medical Research Institute of Infectious Diseases (USAMRIID), 1425 Porter St., Ft. Detrick, MD 21702-5011, USA

## Abstract

**Background:**

The long latent stage seen in syphilis, followed by chronic central nervous system infection and inflammation, can be explained by the persistence of atypical cystic and granular forms of *Treponema pallidum*. We investigated whether a similar situation may occur in Lyme neuroborreliosis.

**Method:**

Atypical forms of *Borrelia burgdorferi *spirochetes were induced exposing cultures of *Borrelia burgdorferi *(strains B31 and ADB1) to such unfavorable conditions as osmotic and heat shock, and exposure to the binding agents Thioflavin S and Congo red. We also analyzed whether these forms may be induced *in vitro*, following infection of primary chicken and rat neurons, as well as rat and human astrocytes. We further analyzed whether atypical forms similar to those induced *in vitro *may also occur *in vivo*, in brains of three patients with Lyme neuroborreliosis. We used immunohistochemical methods to detect evidence of neuroinflammation in the form of reactive microglia and astrocytes.

**Results:**

Under these conditions we observed atypical cystic, rolled and granular forms of these spirochetes. We characterized these abnormal forms by histochemical, immunohistochemical, dark field and atomic force microscopy (AFM) methods. The atypical and cystic forms found in the brains of three patients with neuropathologically confirmed Lyme neuroborreliosis were identical to those induced *in vitro*. We also observed nuclear fragmentation of the infected astrocytes using the TUNEL method. Abundant HLA-DR positive microglia and GFAP positive reactive astrocytes were present in the cerebral cortex.

**Conclusion:**

The results indicate that atypical extra- and intracellular pleomorphic and cystic forms of *Borrelia burgdorferi *and local neuroinflammation occur in the brain in chronic Lyme neuroborreliosis. The persistence of these more resistant spirochete forms, and their intracellular location in neurons and glial cells, may explain the long latent stage and persistence of Borrelia infection. The results also suggest that *Borrelia burgdorferi *may induce cellular dysfunction and apoptosis. The detection and recognition of atypical, cystic and granular forms in infected tissues is essential for the diagnosis and the treatment as they can occur in the absence of the typical spiral Borrelia form.

## Background

The similarity of clinical and pathological manifestations of syphilis caused by *Treponema pallidum *[1] and Lyme disease caused by *Borrelia burgdorferi *[2] is well established. In analogy to *Treponema pallidum, Borrelia burgdorferi *persists in the brain in chronic Lyme neuroborreliosis [3]. How *Borrelia burgdorferi *is able to survive in infected tissues for years or decades is not well understood. Ways for long term survival may be through transformation into more resistant atypical forms and through intracellular localization.

As early as 1905 it was suspected that the classical spiral (vegetative) form was not the only one that spirochetes could assume [1,4]. Transformation of various types of spirochetes into cystic forms through end knob, loop, ring-shaped and spherule formation has since been repeatedly reported [5-10]. Agglomeration of spirochetes into colonies [11-14], enclosing numerous cystic forms, has been observed both *in vitro *and *in vivo *[12].

*Treponema pallidum *and *Borrelia burgdorferi *produce vesicular budding from the membrane, which may become detached. In *Borrelia burgdorferi *these free vesicular or granular structures contain spirochetal surface proteins and linear and circular DNA [15,16].

Granular disintegration of spirochetes resulting in a chain of fine granules also occurs under adverse conditions [17-22]. Minute granules are liberated from the periplasmic sheath through budding and extrusion, which may multiply and may be transmissible [23-31]. Their presence in syphilitic patients was regarded as confirmatory of the syphilitic nature of the lesions even in the absence of classical spiral forms [26,27,30]. These spore-like minute granules (0.1–0.3 μm in diameter) may pass the 0.2 μm "China" filter (32) and can grow into young spirochetes [6,19,25-38]. The newly formed spirochetes are delicate L or metacyclic forms [25,32,39].

These various atypical forms were suggested to be part of a complex developmental cycle, a form of resistance to adverse conditions, and a source for reproduction under more favorable conditions. Reconversion of cystic *Borrelia burgdorferi *into the typical spiral form has been demonstrated *in vitro *and *in vivo *[8,10,31,40].

The occurrence of pleomorphic forms of *Treponema pallidum *in the brain in general paresis and their abundance in juvenile paresis is well documented [6,18,26,41,42].

*Treponema pallidum *may invade virtually all parenchymal and mesenchymal cells, including plasma cells, macrophages, neurons and glial cells [39,40,43]. Atypical and cystic forms of *Treponema pallidum *have been observed both extra- and intracellularly [30]. It has also been described in other spirochetal infections [e.g. [44-46]].

Only limited data are available on the occurrence of atypical, cystic or granular forms of *Borrelia burgdorferi *in infected tissues. Their occurrence has been reported in skin lesions [14], in an *ex vivo *system in tonsil tissue [47] and on silver stained hippocampus section in a patient with concurrent Alzheimer disease (AD) and Lyme neuroborreliosis [48]. Intracellular localization of *Borrelia burgdorferi *was observed in macrophages and keratinocytes in the skin [14] and in neurons and glial cells *in vitro *and *in vivo *[3,49-51].

The goal of the present study was to compare whether atypical and cystic forms of *Borrelia burgdorferi *spirochetes induced in vitro are similar to those occurring in vivo. Three patients with chronic Lyme neuroborreliosis were used in the study. Immunodetection of reactive microglia and astrocytes was also performed to detect neuroinflammation.

## Methods

### Cultivation of Borrelia burgdorferi spirochetes in BSK II medium

*Borrelia burgdorferi *spirochetes strains B31 and ADB1 [3,51-53] were cultivated in BSK II medium [54]. To 500 ml BSK medium (Sigma B 3528) containing 6% rabbit serum (Sigma R-7136) and 7% gelatin (Difco 0143-15-1), 6 mg acetyl muramic acid (Sigma A 3007) and 0.2 g N-acetyl glucosamine (Sigma A8625), Rimactan (Novartis, 420 ul) and Fosfocin (Boehringer Mannheim, 300 ul) were added. The spirochetes were cultivated at 32°C. The pH of BSK II medium was adjusted to pH 7.

To induce atypical spirochete forms 5 ml of cultivated *Borrelia burgdorferi *spirochetes (5 × 10^5^/ml) were exposed to various harmful conditions. Spirochetes were exposed to strong acidic and basic conditions by adjusting the pH of the BSK II medium to pH2 and pH10 using sterile 1 M HCl or 1 M NaOH. The harmful effect of alcohol was analyzed by adding 1 ml of either 70% or 95% ethanol to 5 ml cultivated spirochetes. Heat shock was produced by cultivating spirochetes at 45°C.

Spirochetes are known to bind Congo red and Thioflavin S [55,56], both of which are widely used to stain amyloid. To analyze whether they may induce atypical Borrelia forms, 1 mg or 5 mg of Congo red, or 1 mg or 5 mg Thioflavin S were directly added to 5 ml of spirochete culture. The same amounts dissolved in 2 ml of 70% alcohol were also used to induce atypical spirochetes. The effect of acridin orange, another fluorochrom which binds to spirochetes, was also analyzed by adding 1 mg or 5 mg acridin orange powder to 5 ml of cultivated spirochetes.

Following 1 hour, 6 hours, and 1 week exposure times, 50 μl samples were taken and put on glass slides, cover-slipped, and then examined by dark field microscopy. Series of 50 μl samples were used to prepare smears for histochemical and immunohistochemical investigation. Additional 500 μl samples were removed and fixed in glutaraldehyde for atomic force microscopy (AFM) analysis. Spirochetes cultivated at 32°C at pH 7 for the same periods of time were used as controls. An exception with respect to the exposure times was induction of osmotic shock by cold H2O. Two ml sterile cold H2O was added to 5 ml cultured spirochetes that had been collected by centrifugation at 1000 rpm for 5 minutes. Here the samples were examined following 1 and 6 hours of exposure.

In order to analyze whether the typical spiral Borrelia form may be resuscitated, at the end of each experiments 200 μl samples were reinoculated in BSK II medium at Ph7 and following one week of culture at room temperature, 30 μl samples were analyzed by dark field microscopy.

### Infection of cell cultures with Borrelia burgdorferi

Superior cervical ganglia from 8- to 12-day-old chicken embryos were dissociated as described previously [57]. Briefly, neurons were separated from non-neuronal cells using a density gradient formed with Percoll. The sympathetic neurons were then grown for 3–4 weeks in serum containing medium on a polyornithine substrate pre-coated with heart-conditioned medium [57]. Neurons dissociated from the telencephalon of 21-day-old rat were cultured either on collagen or polylysine substrate in a serum-containing medium [58]. Rat primary astrocytes (10^6^) were prepared as described earlier [59]. Following the characterization of the primary astrocytic cell cultures using anti-GFAP antibody (Dako, Z334) more than 95% of the cells were GFAP reactive (not shown here). The cells were cultured in 2 well chambers (177429 Lab-Tek, Christschurch, New Zealand) or in six well clusters (3506, Costar, Acton, Maryland) in a humidified CO2 (6%) incubator at 37°C.

To infect neurons and astrocytes, Borrelia spirochetes of the virulent strains B31 and ADB1, the latter having been cultured from the brain of a patient with concurrent Lyme neuroborreliosis and AD [3], were employed. Equal volumes of medium for the given primary cells and for spirochetes (BSK II) were used as the culture medium. The final concentration of spirochetes in the infected medium corresponded to 5 × 10^5^/ml. Before exposure to spirochetes, the cells were tested with 4',6-diamidine-2'-phenylindole dihydrochloride (DAPI, 236 276, Boehringer Mannheim, Germany) to exclude Mycoplasma infection. Parallel cultures not infected with spirochetes were always used as controls.

After 1 week exposure, the medium was removed and the cells were rinsed with PBS (2 ml, 2 × 3 minutes). To analyze the morphology of free floating spirochetes, 50 μl samples of culture medium were taken and analyzed by dark field microscopy. Smears were also prepared for histochemical and immunohistochemical analyses.

After 1 week exposure, 200 μl samples from all infected cell cultures were also re-inoculated in BSK II medium and were cultivated at room temperature, Ph 7, for one week. Then 30 μl samples were analyzed by dark field microscopy.

### Detection of apoptosis by deoxynucleotidyltransferase (TdT)-mediated dUTP nick end labeling (TUNEL)

Cells in 6 wells chambers were fixed with 4% paraformaldehyde for 10 minutes in room temperature. Following an incubation with proteinase K (20 μg/ml) in TRIS HCL (pH 7.4) for 15 minutes at 37°C the cells were rinsed with 2 ml PBS (2 × 3 minutes). Then cells were treated with a permeabilisation solution containing 0.1% Triton X100, in 0.1% sodium citrate, for 2 minutes on ice followed by a rinse with PBS (2 × 3 minutes).

The cells were incubated with a freshly prepared TUNEL reaction mixture kept on ice containing 45 μl of TUNEL label solution (1767291, Boehringer) containing unlabeled dNTPs and fluorescein isothiocyanate tagged dUTP (FITC-dUTP) and 5 μl of TUNEL enzyme (Terminal deoxynucleotidyl Transferase (TdT) (1767305, Boehringer) for 1 hour and 30 minutes at 37°C in a humidified chamber. For a negative control the TUNEL enzyme was omitted from the TUNEL reaction mixture and 50 μl TUNEL label solution alone was used.

### Detection of pleomorphic Borrelia forms in vivo

Brains of three patients with pathologically and serologically confirmed Lyme neuroborreliosis and concurrent AD were analyzed [3]. From the brains of these three patients, aged 74, 78, and 86 years, spirochetes were successfully cultivated in BSK II medium. In two of them (strains ADB1 and ADB2) 16SrRNA gene sequence analysis identified the spirochetes as *Borrelia burgdorferi sensu stricto (s. s.)*.

To detect whether atypical and cystic forms of *Borrelia burgdorferi *spirochetes are present in the brains of these patients, frozen sections of the hippocampus, frontal, temporal and parietal cortex were analyzed using dark field microscopy, as well as histochemical and immunohistochemical techniques. Before immunostaining the sections were fixed in acetone for 10 minutes at 4°C. Acetone fixed frozen sections which were cut from samples taken from identical brain areas in three control patients without neurological symptoms and without brain lesions were also processed and analyzed in the same fashion.

Paraffin sections (20 μm thick) cut from various cortical samples (from archival material of the Armed Forces Institute of Pathology, USA) of two patients (31 and 52 year old males) with clinically, serologically and pathologically confirmed general paresis, were also analyzed for the presence of *Treponema pallidum*. The goal was to compare atypical spirochetal forms of *Borrelia burgdorferi *in Lyme neuroborreliosis with those of *Treponema pallidum *in general paresis.

### Dark field microscopy, histochemical and immunohistochemical analysis of spirochetes

From cultivated Borrelia spirochetes exposed to various harmful conditions, 50 μl samples were used as wet preparation for dark field microscopy analysis. Additional samples (50 μl) were used to prepare smears for the histochemical and immunohistochemical analyses. In order to analyze free floating spirochetes in Borrelia infected cell cultures, 50 μl samples of the co-culture medium were analyzed.

Smears and brain sections were stained with the Warthin-Starry and Bosma-Steiner silver techniques as described for the detection of spirochetes. Spirochetal DNA was detected in smear preparations of cultivated spirochetes exposed to various adverse conditions, in infected cells, and in unfixed frozen brain sections by staining with DAPI (236 276, Boehringer Mannheim, Germany) following instructions of the manufacturer. The same preparations were also used for immunohistochemical analysis. Smears prepared on glass slides from cultivated spirochetes, from medium of infected cells, and from cryostat cut brain sections were fixed in acetone for 10 minutes at 4 °C prior to immunostaining. Infected cells in six wells, where acetone cannot be used, were fixed in 4% paraformaldehyde for 5 minutes. Before immunostaining, frozen brain sections following acetone fixation were incubated in 0.1% amylase for 5 min at 37°C. The following anti-*Borrelia burgdorferi *antibodies were used: monoclonal anti-OspA (H5332, H3T5, Symbicom, 1:50) and anti-flagellin (G 9724, H605, Symbicom, 1:50), polyclonal B65302R (Biodesign, 1:100) and BB-1017 (1:500) [3] antibodies. The specificity of these mono- and polyclonal antibodies was previously tested by Western blot analysis [3].

For immunostaining, the avidin-biotin-peroxidase technique was used. Following 24, 48 or 72 hours incubation with a primary antibody at 4°C, the sections were incubated with the appropriate secondary antibodies. For the monoclonal antibodies, a biotinylated F(ab) fragment of affinity isolated rabbit anti-mouse immunoglobulin (Dako, E413) was used. The immunoreaction was revealed by diaminobenzidine (DAB) alone, or with nickel-ammonium sulfate as described previously [60]. Frozen sections immunostained in the absence of a primary antibody or with an irrelevant mono- or polyclonal antibody were used as negative controls. Immunostaining was also performed with various anti-*Borrelia burgdorferi *antibodies using FITC tagged anti-mouse or anti-rabbit secondary antibody depending on the primary antibody used. The green fluorescence of *Borrelia burgdorferi *spirochetes was analyzed with a Zeiss fluorescent microscope. A monoclonal antibody (Biogenesis 7263-1006 or Chemicon MAB995, dil.1: 200) for the analysis of the presence of bacterial peptidoglycan, a bacterial cell wall component of virtually all Eubacteria, including spirochetes, was also used as previously described in detail [61].

Floating paraffin sections (20 μm thick) of the cerebral cortex of the two patients with general paresis were immunostained with a polyclonal anti-*Treponema pallidum *antibody (Biodesign, B65210R).

### Detection of neuroinflammation

Paraffin sections of the hippocampus, frontal and parietal cortices of the three patients with Lyme neuroborreliosis were also used for the immunohistochemical detection of reactive microglia and astrocytes. Anti-HLA-DR (clone CR3/43, M775, Dako) and anti-CD68 (clone KP1, M814, Dako) monoclonal antibodies were used to visualize microglia and a polyclonal anti-GFAP antibody (Z334, Dako) to detect astrocytes. Paraffin sections of the same cortical areas of a female patient (aged 59 years) without brain lesion were used as controls. In addition, sections of the three patients with Lyme neuroborreliosis were also immunostained with the omission of these primary antibodies.

### Atomic force microscopy (AFM) analysis

To 500 μl samples of cultivated Borrelia spirochetes exposed to various harmful conditions 500 μl of 2.5% buffered glutaraldehyde was added. Samples were then stored at 4°C until used for the atomic force microscopy (AFM) analysis. 20–50 μl samples were put on the surface of a Nucleopore^® ^filter of 2 μm hole size and were dried at room temperature in air, as previously described [62]. The filters were fixed on metallic discs or on glass slides using a double face rubber strip, and were imaged with a Bioscope I atomic force microscope (AFM) and a Nanoscope^® ^III atomic force microscope (AFM) equipped with a J-scanner. All the images were taken in the tapping mode at room temperature in air. The scanning rate varied from 0.1 to 5 Hz. Images were obtained in both the constant force mode providing true height, and the amplitude mode, for highlighting sharp contours. The images were processed and the measurements were done using the Nanoscope III image processing software.

The human brains analyzed were from the University Institute of Pathology, Lausanne, Switzerland. The study adhered to the tenets of the Helsinki Declaration. Animal experimentation conformed to the Guide for the Care and Use of Laboratory Animals, formulated by the National Research Council, 1996, and the Swiss law on animal protection.

## Results

**Figure 1:**
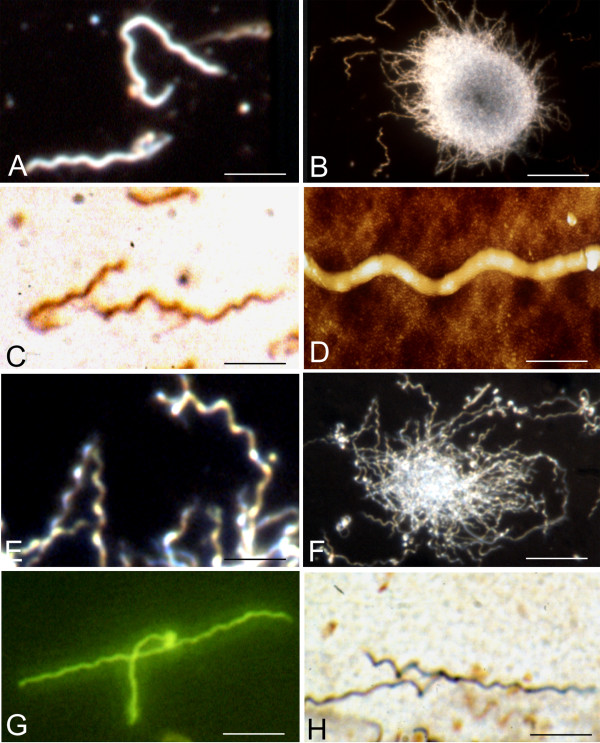
**Characteristic morphology of *Borrelia burgdorferi *seen by various techniques following one week of culture in BSKII medium.** A and B: Dark field microscopy images of *Borrelia burgdorferi *strain B31 showing the usual spiral form of spirochetes (A) and their agglomeration into colony-like masses (B). Similar spiral morphology of strain B31 is illustrated by OspA immunoreactivity (C) and by atomic force microscopy (AFM) imaging (D). E and F: Dark field microscopy images showing the typical spiral form (E) and colony formation (F) of *Borrelia burgdorferi *strain ADB1. G: Similar spiral morphology of strain ADB1 shown by immunostaining with a polyclonal anti-*Borrelia burgdorferi *antibody (Biodesign, B65302R). The green fluorescent immunoreaction was revealed with an FITC tagged secondary antibody. H: Similar morphology of strain ADB1 revealed by silver impregnation with the Bosma Steiner microwave technique. Bars: A, C = 10 μm; B = 30 μm; D = 1 μm; E, G, H = 8 μm; F = 25 μm.

Figure 1 illustrates the typical morphology and colony-like formation of strains B31 (A-D) and ADB1 (E-H). Panels A and B show the regular coiled morphology and colony-like aggregates of spirochetes (B31 strain) as observed by dark field microscopy analysis. Regularly coiled OspA-immunoreactive spirochetes of the same strain are seen following immunostaining with a monoclonal anti-OspA antibody in panel C. An atomic force microscopy (AFM) image of a regularly coiled fragment of a Borrelia spirochete (strain B31) is visible in panel D. The typical coiled morphology of spirochetes of the ADB1 strain, which were cultivated from the brain of a patient with chronic Lyme neuroborreliosis, is illustrated in panels E and F by dark field microscopy and in panel G by immunohistochemistry using a polyclonal anti-*Borrelia burgdorferi *antibody (Biodesign, B65302R). The primary anti-*Borrelia burgdorferi *antibody was revealed with FITC-tagged secondary antibody showing green fluorescence. Panel H illustrates the typical spiral appearance of spirochetes (strain ADB1) by silver impregnation using the Bosma-Steiner microwave technique.

The typical spiral forms of Borrelia spirochetes were converted to atypical and cystic forms when exposed to various unfavorable culture conditions. Atypical and cystic forms were seen at 1 hour exposure. Their numbers increased following 6 hours exposure and was highest at 1 week, where the majority of spirochetes showed atypical morphology. Osmotic shock induced by cold sterile distilled water induced atypical and cyst forms of the majority of spirochetes following 1 and 6 hour exposure. The atypical and cyst forms retained an affinity for silver and were immunoreactive with the various anti-*Borrelia burgdorferi *antibodies.

The morphological changes were similar in both the B31 and ADB1 strains, whether induced by osmotic shock, heat shock, alcohol, acidic or basic pH. Atypical and cystic forms were also seen following Congo red and Thioflavin S exposures.

A stronger effect was observed when Thioflavin S and Congo red were dissolved in ethanol before addition to the culture.

Atypical forms included knob, ring-shaped and loop formations, uni- or multi-spirochetal cysts, bleb formation, granular disintegration and colony-like masses enclosing numerous cystic forms. Following 1 week exposure, free minute granules and re-growing of slender motile L forms of young spirochetes along injured spirochetal cells were observed.

**Figure 2:**
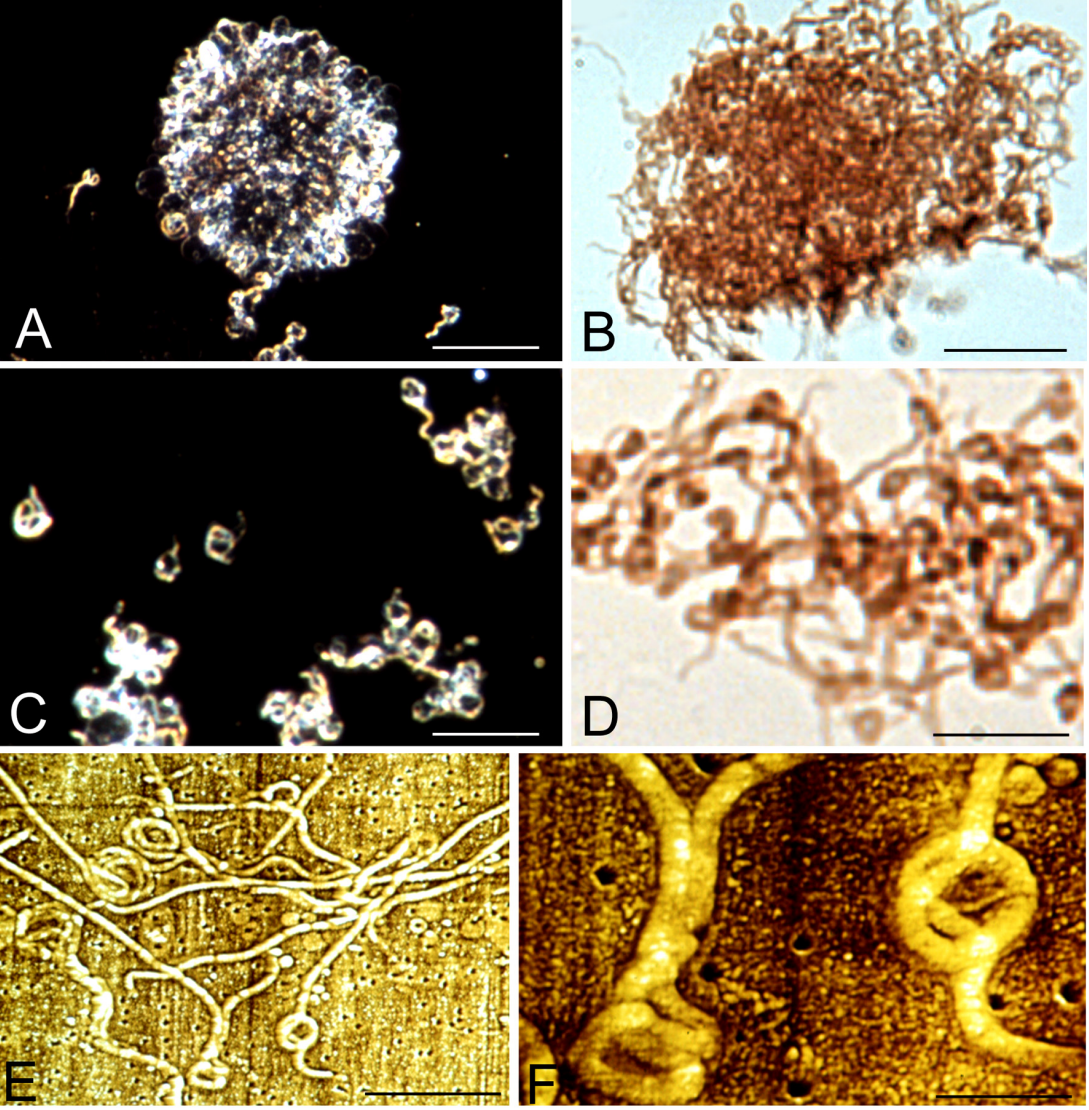
**Atypical forms of *Borrelia burgdorferi *(B31 strain) spirochetes induced by harmful culture conditions. **A-D: Large agglomerates of atypical ring shaped and spherule forms of *Borrelia burgdorferi *after one week of BSKII culture followed by 5 minutes of osmotic shock generated by cold distilled water. A and C: Low and high power fields as revealed by dark field microscopy. B and D: Similar morphology in low and high power fields as revealed by immunohistochemistry using the anti-OspA monoclonal antibody. E and F: Low and high power atomic force microscopy (AFM) images showing similar morphology. In this case the inducing agent was 1 mg of Thioflavin S added to the medium at the commencement of one week of culture. See materials and methods for details. Bars: A, B = 30 μm, C-D = 20 μm; E = 5 μm, F = 1 μm.

Figure 2 illustrates rolled spirochetes forming ring-shaped and globular structures. Panel A-D show large aggregates of multiple rolled, ring-shaped and cystic spirochetes in response to osmotic shock generated by cold sterile distilled water. Panels A and C illustrate these atypical forms as seen by dark field microscopy at lower (A) and higher (C) magnifications. Panels B and D illustrate similar results following immunostaining with a monoclonal anti-OspA antibody. Atomic force microscopy (AFM) images of loop and ring formations of Borrelia spirochetes are illustrated in panels E and F.

**Figure 3:**
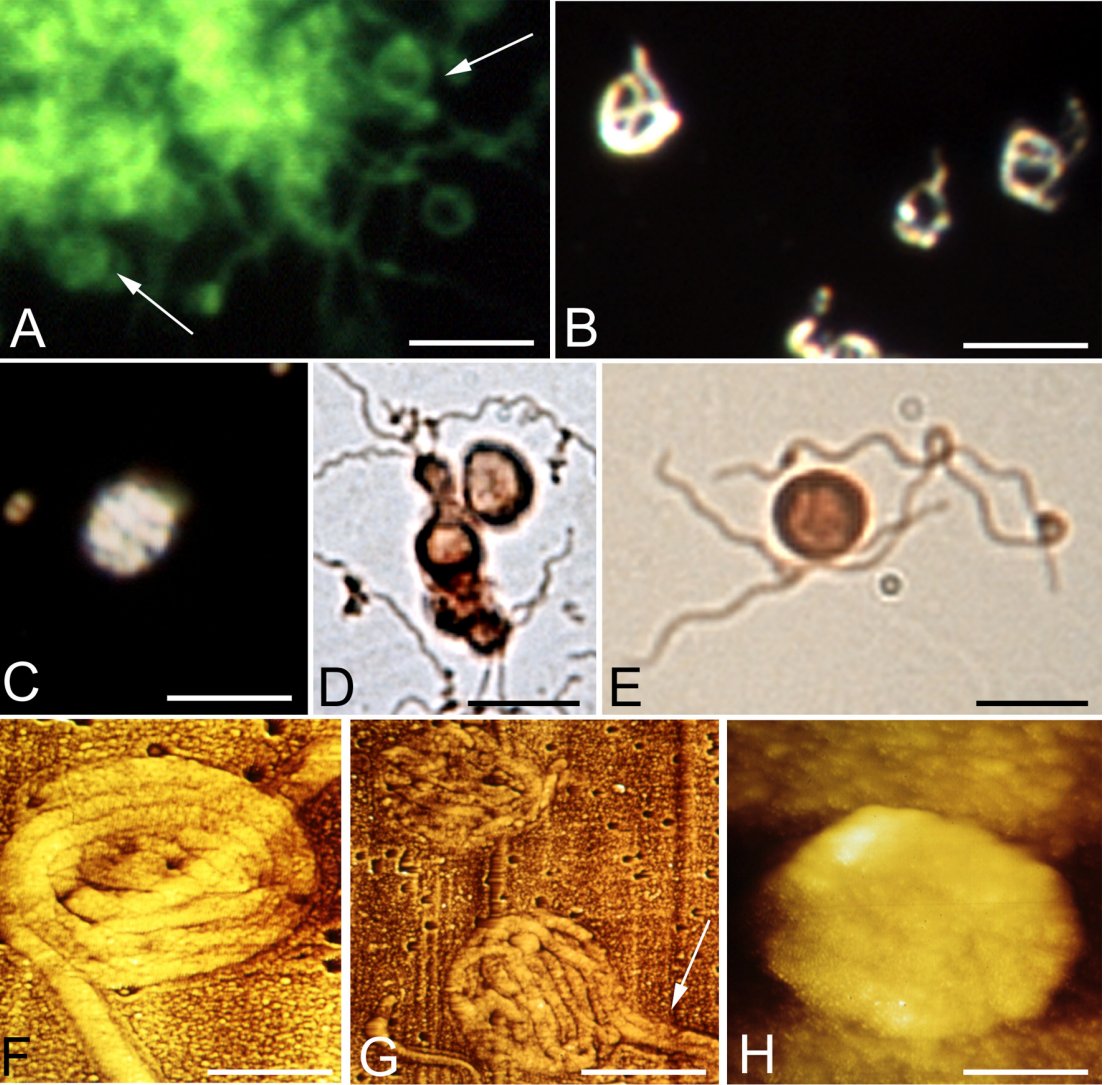
**Rolled and cystic forms of *Borrelia burgdoferi *spirochetes observed after one week of culture in medium to which Thioflavin S had been added.** A: Observation by Thioflavin S fluorescence. Arrows point to rolled cystic forms at the periphery of an agglomerated mass of spirochetes from strain B31. Rolled (B) and cystic (C) forms observed by dark field microscopy (strain B31). D and E: Cyst forms of *Borrelia burgdorferi *(strains ADB1 and B31, respectively) following immunostaining with the monoclonal anti-OspA antibody. F-H: Atomic force microscopy (AFM) images of Borrelia cysts. Rolled spirochetes are clearly visible in F (strain B31) and G (strain ADB1). Arrow in G shows that the cyst is formed by two spirochetes rolled together. H: The cystic form is entirely covered by a thickened external membrane masking the content of the cyst (strain B31). Bars: A-D = 6 μm; E = 5 μm; F = 1 μm; G = 2.5 μm; H = 0.5 μm.

Figure 3A illustrates atypical and cystic forms of *Borrelia burgdorferi *induced by Thioflavin S (1 mg dissolved in 2 ml 70% ethanol). The agglomeration of spirochetes exhibits green fluorescence due to the binding of Thioflavin S. Numerous ring-shaped and globular cystic forms (arrows) were observed in the periphery of the spirochetal mass. Similar rolled and cystic Borrelia forms (B, C) induced by Thioflavin S are illustrated by dark field microscopy. Panels D and E show spirochetes with similar morphology, which were immunostained with anti-OspA antibody. Panels F-H show the results of atomic force microscopy (AFM) analysis. The atomic force microscopy (AFM) images reveal rolled spirochetes inside of cysts. Cystic forms entirely covered by a thickened external membrane masking the content were also observed. One of them is illustrated in panel H. It is similar to those observed by dark field microscopy as well as with an anti-OspA antibody (compare H with C, D and E).

Atypical cystic, granular forms and colony-like aggregation of spirochetes into large masses enclosing cystic forms were also observed following 1 week infection of primary neurons and astrocytes with Borrelia spirochetes. The morphological changes observed were similar in chicken sympathetic neurons, rat telencephalic neurons and also in rat and human primary astrocytes. Both Borrelia strains (B31 and ADB1) showed identical morphological changes. Following 1 week exposure of the cells to *Borrelia burgdorferi*, it is difficult to find spirochetes which have preserved the typical spiral form.

**Figure 4:**
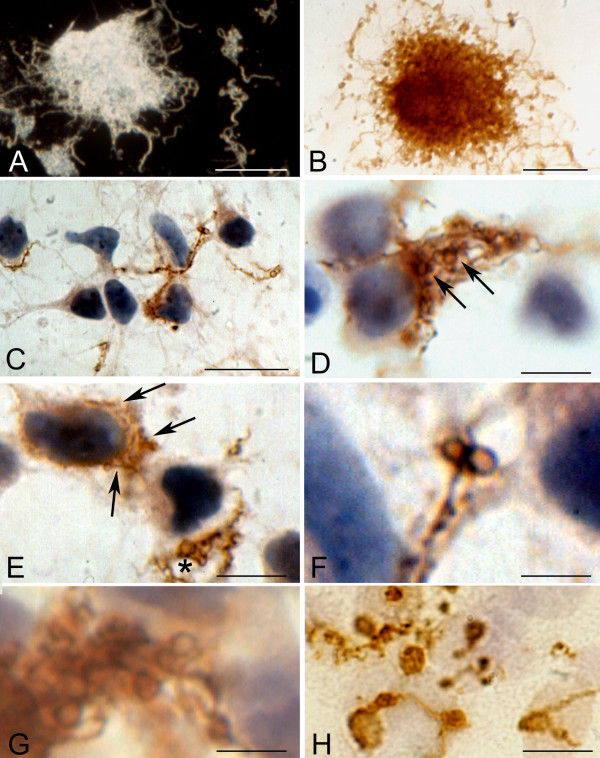
**Atypical and cystic Borrelia forms following 1 week exposure of primary neuronal and astrocytic cultures to *Borrelia burgdorferi*.** Panels A, C-G illustrate atypical Borrelia forms in primary chicken neuron cultures and panels B and H in rat astrocytic cultures. A is by dark field microscopy; B-H are by anti-OspA immunostaining. A: Formation of large colony like aggregates in a neuronal culture as observed by dark field microscopy (strain B31) and in astrocytic culture as visualized by anti-OspA immunostaining (strain ADB1). C: OspA positive Borrelia spirochetes closely surrounding neurons (strain B31). D: Atypical filamentous and ring-shaped cystic, apparently intra-cellular spirochetes in a neuron (strain B31). E: Filamentous and granular forms are seen in the cytoplasm in one neuron. Some extracellular spirochetes show ring-shaped atypical forms (strain ADB1). F: Immunoreactive ring-shaped spherules are seen at one end of a spirochete with some small minute granules along the injured cell (strain B31). G: A small colony like mass is seen in which numerous ring-shaped spherules are visible in the absence of typical coiled spirochetes (strain B31). In H ring-shaped and cystic forms in infected rat astrocytic culture are visible (strain ADB1). Bars: A = 40 μm; B = 30 μm; C = 60 μm; D, E = 10 μm; F-H = 5

Figure 4 illustrates atypical forms of spirochetes following 1 week exposure of chicken primary sympathetic neurons (A, C-G) and rat astrocytes (B and H) to *Borrelia burgdorferi*, strain ADB1. Large colony-like aggregates are illustrated in infected neuronal culture (A) as seen by dark field microscopy and in rat primary astrocytic cultures (B) immunostained with an anti-*Borrelia burgdorferi *antibody. Borrelia spirochetes surrounding neuronal perikarya are seen in panel C. Panels D and E illustrate OspA immunoreactive intracytoplasmic atypical filamentous, ring shaped (arrow) forms of *Borrelia burgdorferi*. Some extracellular spirochetes showing ring-shaped formation are also present (E, arrow). Panel F shows an OspA immunoreactive atypical spirochete with a double ring-shaped formation at one end and some OspA positive granules along the injured spirochete. A small colony like mass is illustrated in panel G in which the majority of spirochetes show atypical ring-shaped cystic formations. Regularly coiled spirochetes are not present. OspA immunoreactive ring shaped forms and spherules of Borrelia spirochetes in rat astrocytic culture are illustrated in panel H.

**Figure 5:**
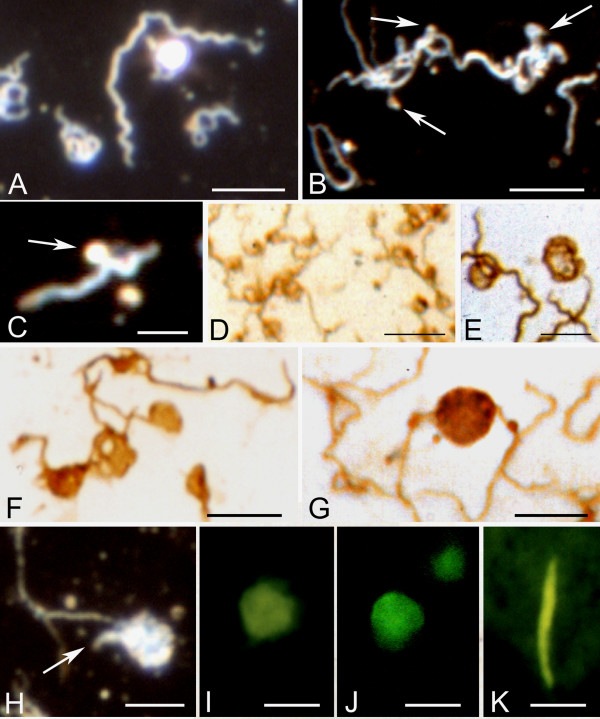
**Atypical cystic spirochetes in the medium of neuronal and astrocytic cultures following 1 week exposure to *Borrelia burgdorferi*.** A-C and H are dark field microscopy images. Panels D-G illustrate immunostaining with anti-OspA antibody. A: In addition to typical spiral-shaped spirochetes, several rolled, looped, and ring-shaped forms are seen. B: Atypical spirochetes showing ring shaped forms, blebs still attached to the spirochetes (arrows) as well as some minute granules. C: Arrow points to a bleb still attached to the surface of the spirochete. Multiple ring-shaped (D, E) and cystic forms (F, G) are visible. Notice that in G the cyst is formed by two spirochetes. H-J: Borrelia cysts as visualized by dark field microscopy; the arrow points to the end of the spirochete forming the cyst. Cyst form as seen by immunofluorescence using anti-OspA antibody (I) and DAPI-DNA staining (J). K: OspA immunoreactive thick, elongated bodies were also observed. Panels A-G correspond to strain ADB1 and H-K to strain B31. Bars: A, B = 10 μm; C = 4 μm; D = 8 μm; E, F = 6 μm; G = 5 μm; H-K = 4 μm.

Atypical forms, including ring-shaped, uni- or multi-spirochetal cystic and granular forms also occurred free floating in the medium of primary neuronal and glial cell cultures. Figure 5 illustrates these atypical forms following 1 week exposure to Borrelia spirochetes. Dark field microscopy images of ring-shaped and cystic forms are seen in panels A and B. Arrows in B and C point to bleb formations still attached with thin stalks to the surface of the spirochete cell. Panels D-G show OspA immunoreactive multiple ring-shaped (D and E) and cystic (F-G) Borrelia forms. The cysts may sometimes be formed by multiple spirochetes. For example, in panel G an anti-Ospa immunoreactive cyst formed by two spirochetes is visible. Panels H-J illustrate spirochetal cysts as visualized by dark field microscopy (H), by immunofluorescence using an anti-OspA antibody (I) and by DAPI staining (J). OspA immunoreactive large, thick, elongated bodies were also observed as seen in panel K. Panel H shows dark field microscopy image of a cyst where the free end (arrow) of the rolled spirochete is visible.

**Figure 6:**
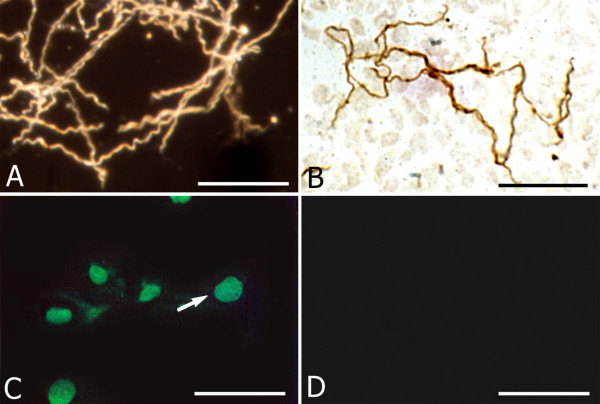
**Recovery of the typical vegetative form of spirochetes re-cultured in BSK II medium and nuclear fragmentation of rat primary astrocytes exposed to *Borrelia burgdorferi*.** A: Dark field microscopy image of numerous *Borrelia burgdorferi *spirochetes (B31 strain) exhibiting the regular spiral form, re-covered in BSK-II medium following 1 week exposure to 5 mg Thioflavin S. B: Typical vegetative form re-covered from rat astrocyte culture exposed to *Borrelia burgdorferi *(ADB1) for 1 week, as revealed with a rabbit polyclonal anti-*Borrelia burgdorferi *antibody (BB-1017). Compare the regular spiral morphology of these spirochetes with those seen in Fig. 4H, where virtually all spirochetes showed atypical forms. C: Green fluorescent apoptotic nuclei of rat astrocytes as visualized with the TUNEL technique using FITC tagged dUTP. D: Uninfected primary astrocytes cultivated in parallel for 1 week did not show nuclear fragmentation. Bars: A, B: 25 μm; C, D: 50 μm.

When spirochetes were re-cultured from various harmful conditions and from infected cell cultures in BSK II medium in optimal condition, the typical spiral form of Borrelia spirochetes was recovered. A dark field microscopic image of the vegetative form of Borrelia spirochetes recovered from Thioflavin S (5 mg) treated cultures is illustrated in Figure 6A. Classical spiral Borrelia spirochetes recovered from infected rat astrocytes cultured for 1 week are illustrated in Figure 6B by their immunoreaction to anti-Borrelia antibody BB-1017.

Figure 6C illustrates nuclear fragmentation in astrocytes following 1 week exposure to *Borrelia burgdorferi *as visualized by the TUNEL method using FITC tagged dUTPs. Nuclear fragmentation was not observed in control cultures, which were not infected with Borrelia (D).

**Figure 7:**
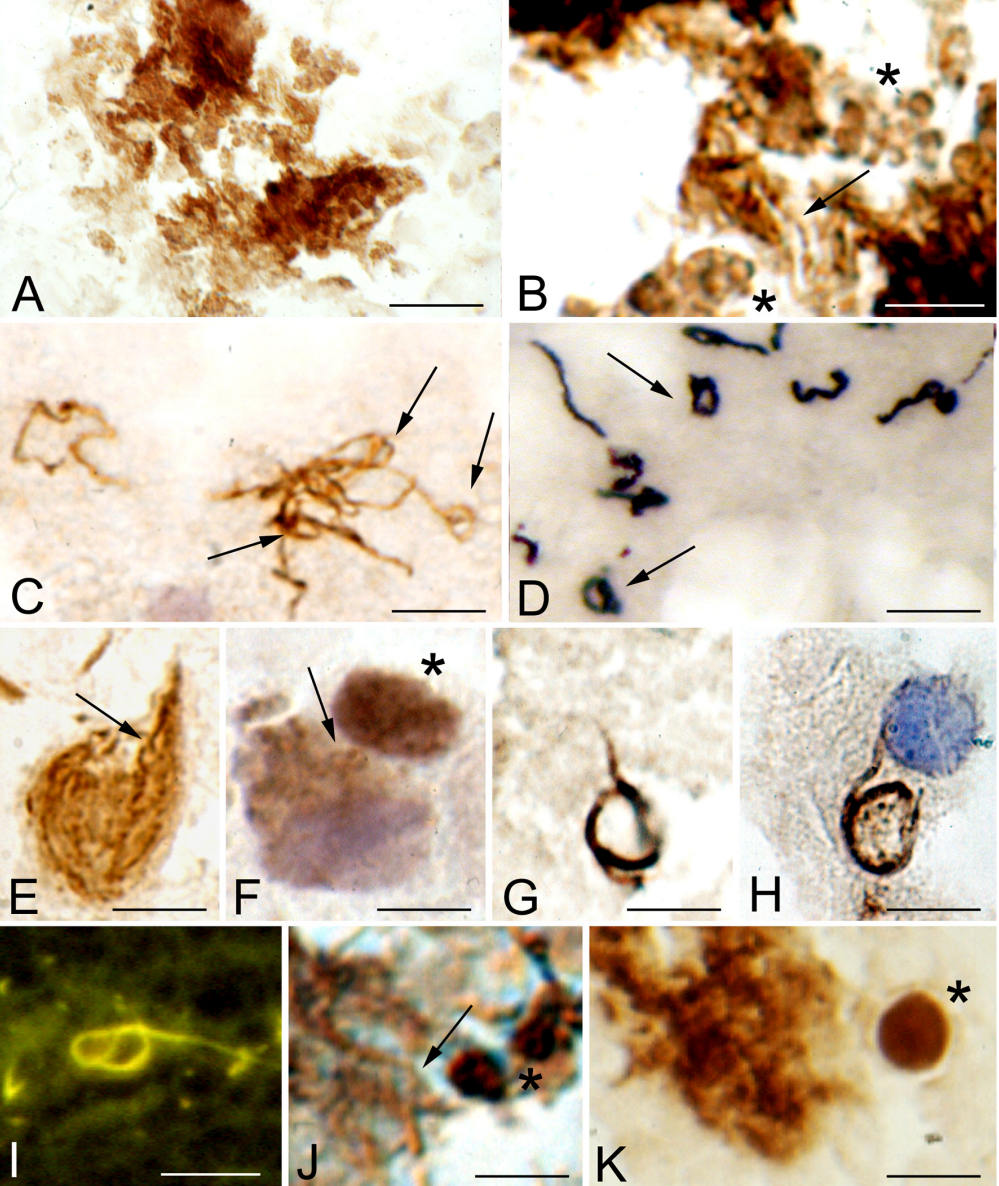
**Extra- and intracellular atypical and cystic forms of spirochetes in the cerebral cortex of a patient with pathologically and serologically confirmed chronic Lyme neuroborreliosis where *Borrelia burgdorferi sensu stricto *was cultivated from the brain.** A: Colony-like agglomeration of spirochetes as revealed by monoclonal anti-OspA antibody in the cerebral cortex. B: A close up of the central part of the mass seen in A. In addition to a few helically shaped spirochetes (arrow) numerous ring-shaped forms and spherules (asterisk) are visible, which are identical to those observed *in vitro *following 1 week Borrelia exposure of primary neurons (compare with Fig. 4G). C: Spirochetes showing loop or ring-shaped formations (arrows) in the cerebral cortex immunostained with a polyclonal anti-*Borrelia burgdorferi *antibody (Biodesign, B65302R). They are similar to those of *Treponema pallidum *(arrows in D) observed in the cerebral cortex of a patient with general paresis. Immunostaining was performed using a polyclonal anti-*Treponema pallidum *antibody (Biodesign, B65210R). E: Helically shaped OspA immunoreactive spirochetes in the cytoplasm of a cortical pyramidal neuron. In addition to one more typical form (arrow), fine OspA positive minute granules along filamentous forms are seen. F: Intracellular ring-shaped forms (arrow) showing positive immunoreaction with a polyclonal anti-*Borrelia burgdorferi *antibody (BB1017). They are identical to those observed in chicken primary neurons infected with Borrelia (compare F with Figure 4D). Near the asterisk a large strongly immunoreactive cyst form is visible. Spirochete forming loop in the cerebral cortex (G) and in the cytoplasm of an epithelial cell of the choroid plexus (H) are seen as visualized by anti-OspA and anti-bacterial peptidoglycan antibodies, respectively. I: A similar atypical spirochete forming loops in the cerebral cortex as visualized with Thioflavin S. J: In an area with colony-like spirochete aggregation in addition to some typical, regularly coiled Borrelia spirochetes (arrow) OspA positive cystic forms (asterisk) are seen. K: In the cerebral cortex near the colony-like spirochetal agglomerate a spirochete cyst (asterisk) similar to that observed *in vitro *is visible (compare it with Figure 5 G-J). Immunostaining was performed using a monoclonal anti-OspA antibody. Bars: A = 20 μm; B-J = 10 μm, K = 5 μm. Panels C and E were reprinted from panels F and D of Figure 5 of Mikossy et al., 2004 [3], with permission from IOS Press.

Identical atypical and cystic forms were observed in the cerebral cortex of the three patients with pathologically confirmed chronic Lyme neuroborreliosis. Figure 7 illustrates these atypical and cystic forms. OspA immunoreactive colony-like agglomeration of spirochetes is seen in panel A. In such "colonies" or agglomerates of spirochetes, atypical, stretched filamentous forms, as well as numerous ring-shaped forms and spherules are frequently present. Panel B shows, in the periphery of such agglomerates, atypical ring shaped structures and spherules (asterisks), which are identical to those observed *in vitro*. Ring shaped spirochetes showing a positive immunoreaction with anti-*Borrelia burgdorferi *antibody in the cerebral cortex in a case of parenchymatous Lyme neuroborreliosis are seen in panel C. These ring-shaped forms are similar to those of *Treponema pallidum *(arrows in D) as illustrated in the cerebral cortex of a patient with general paresis using a polyclonal anti-*Treponema pallidum *antibody (Biodesign, B65210R). Arrows point to helical (E) and ring-shaped OspA immunoreactive forms (F) accumulated in the cytoplasm of cortical neurons. Rolled spirochetes forming large rings in the cerebral cortex (G) and in the cytoplasm of an epithelial cell of the choroid plexus (H) are seen in panels G and H, as visualized by anti-OspA and antibacterial peptidoglycan antibodies, respectively. Panel I shows similar atypical rolled forms as visualized with Thioflavin S in the brain of the same patient. In addition to some filamentous spirochete forms with more regular spirals (arrow) cystic forms were also observed in the cerebral cortex (asterisks in J and K). The atypical and cystic spirochetes observed in the brain of the patient from which ADB1 strain was cultivated were identical to those induced when the spirochetes of this strain were cultivated under various harmful conditions, or when primary astrocytes or neurons were infected by these spirochetes. Cortical sections of control cases immunostained with various anti-*Borrelia burgdorferi *antibodies were negative.

**Figure 8:**
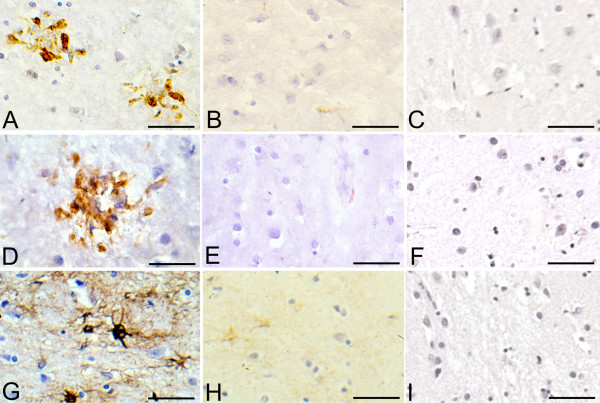
**Chronic neuroinflammation in the frontal cortex of a patient with Lyme neuroborreliosis.** First column (A, D and G): Accumulation of HLA-DR (A) and CD68 (D) immunoreactive microglia forming clumps, and GFAP (G) positive large reactive astrocytes in the frontal cortex of a patient with Lyme neuroborreliosis. Second column (B, E, H) : On frontal sections of the control patient, activated microglia or astrocytes are not visible. Some resting microglia showing weak HLA-DR immunostaining (B), absence of CD68 immunoreaction (E) and weak GFAP immunostaining of non reactive astrocytes and astrocytic processes (H) are visible. C, F and I: Absence of immunoreaction on sections of a patient with Lyme neuroborreliosis where immunostaining was performed with omission of the anti-HLA-DR (C), anti-CD68 (F) and anti-GFAP (I) antibodies. Bars: A, B, F, H, I = 150 μm; C, D, E = 120 μm; G = 100 μm.

There was no apparent lympho-plasmocytic infiltrates on Hematoxylin and Eosin-stained sections in the brains of the three patients with Lyme neuroborreliosis (not shown). However on sections immunostained with monoclonal antibodies for HLA-DR and CD68, abundant reactive microglia, frequently forming clumps, were observed in the cerebral cortex. Accumulation of GFAP positive reactive astrocytes was also present. Figure 8 illustrates HLA-DR and CD68 immunoreactive microglia and GFAP-positive reactive astrocytes in the frontal cortex in one patient, where *Borrelia burgdorferi *(ADB1 strain) was cultivated from the brain. Some HLA-DR reactive resting microglial cells were observed in the frontal cortex of the control patient. No apparent immunostaining was observed with the anti-CD68 antibody. On GFAP-immunostained sections some astrocytes with poor cytoplasm and thin processes without signs of hyperplasia or hypertrophy were visible. Brain sections immunostained with the omission of the primary antibodies were negative.

## Discussion

*Treponema pallidum *and *Borrelia burgdorferi *are associated with various chronic neuro-psychiatric disorders. *Treponema pallidum *persists in the brain and causes various neuropsychiatric disorders including dementia, cortical atrophy and amyloid deposition years or decades following the primary infection [63-65]. The persistence of more resistant atypical cystic and granular forms of *Treponema pallidum*, which are less sensitive to chemicals and antibiotics, are responsible for the long latent stage in chronic syphilis and for the infectivity of tissues devoid of the demonstrable vegetative form of spirochetes. The intracellular localization of *Treponema pallidum *is another way of evading from destruction by the host immune system [30,39]. Virtually all types of mammalian cells can be invaded by *Treponema pallidum *resulting ultimately in functional cell damage and cell destruction.

Recently we reported evidence that *Borrelia burgdorferi *can also persist in the brain in chronic Lyme neuroborreliosis and, in analogy to *Treponema pallidum*, may cause dementia, cortical atrophy and amyloid deposition [3,49,51]. Only limited data have previously been available on the presence of atypical, cystic forms of spirochetes in the brain in chronic Lyme neuroborreliosis. Whether such forms may eventually cause functional damage and cell death is still not certain.

Here we analyzed atypical, cystic forms of *Borrelia burgdorferi *induced by unfavorable culture conditions and compared these with forms observed following 1 week of infection of primary chicken and rat neurons, as well as primary rat and human astrocytes. We also analyzed whether similar atypical and cystic forms may occur *in vivo *in brains of patients with pathologically and serologically confirmed Lyme neuroborreliosis and compared them to the atypical forms of *Treponema pallidum *in brains of patients with general paresis. The results show that under harmful culture conditions, the typical forms of Borrelia spirochetes are replaced by atypical forms varying from ring-shaped and cystic forms to fine single granules of almost submicroscopic size. These results are in harmony with previous observations [8,55,66]. The effect of osmotic shock induced with cold distilled water or heat shock was identical to those previously observed in other spirochetes [25,67]

Thioflavine S and Congo red had a similar effect. The mechanism of the harmful effect of these dyes is not known. They may act by binding to the outer sheath of Borrelia spirochetes [e.g. [56]]. Thioflavin S and Congo red are widely used to detect amyloid deposits in affected tissues. Several observations suggested that *Borrelia burgdorferi *possesses amyloidogenic proteins [51,68,69]. Peptides derived from the OspA single-layer beta-sheet showed fibrillary amyloid formation, which may be an explanation of the binding of Thioflavin S and Congo red to the outer surface of *Borrelia burgdorferi*.

Atomic force microscopy (AFM) analysis showed rolled Borrelia spirochetes inside of a cyst covered by a thin outer membrane. This has also been observed in various types of spirochetes [e.g. [11,38,70]] including *Borrelia burgdorferi *[8] by transmission electron microscopy analyses. Uni- or multi-spirochetal cysts may be formed. We illustrated by atomic force microscopy (AFM) rolling of two Borrelia spirochetes to form a cyst. The size of such cysts depends on the number of spirochetes packed inside of the cyst [8]. We observed bleb formation, connected to Borrelia spirochetes by a fine stalk, in both Borrelia strains. Thin newly formed spirochetes attached to spirochete cells, and to free minute granules were also observed.

Similar atypical, cystic and granular forms were observed in primary neuronal and astrocytic cell cultures exposed for 1 week to the *Borrelia burgdorferi *strains B31 and ADB1. Nuclear fragmentation of a subset of infected cells as revealed by TUNEL suggests that *Borrelia burgdorferi *can cause functional damage and cell death. The intracellular localization of filamentous, ring-shaped, cystic and granular forms suggests that such intracellular Borrelia spirochetes can be protected from destruction by the host immune system.

Identical atypical and cystic forms were also observed in the cerebral cortex of the three patients with chronic Lyme neuroborreliosis with concurrent AD. This indicates that *Borrelia burgdorferi *spirochetes can form resistant cystic forms, which may persist in the brain. Numerous colonies were also observed *in vitro *and *in vivo*. In the brain they were restricted to the cerebral cortex. These spirochetal masses included numerous cystic forms as has been described for *Treponema pallidum *and other spirochetes. Like for *Treponema pallidum *in neurosyphilis, atypical and cystic forms of *Borrelia burgdorferi *were also observed intracellularly in the brains of these patients, as it has previously been documented [3,51]. These results suggest that *Borrelia burgdorferi*, in analogy to *Treponema pallidum *may also invade neurons and glial cells and cause cell dysfunction and progressive cell death.

The results also showed that atypical Borrelia forms may be present in the absence of typical coiled forms, indicating that detection of atypical forms in infected tissues may be of diagnostic value. *Treponema pallidum *and *Borrelia burgdorferi *can persist in infected tissues, even in the absence of an apparent lymphoplasmacytic infiltration. Consequently, when the clinical and histopathologic features suggest syphilis or Lyme disease, the detection of these spirochetes in infected body fluids and tissues may be of diagnostic importance [55,71].

*Borrelia burgdorferi *cultured in harmful conditions and in infected cell cultures where virtually all spirochetes showed pleomorphic and cystic forms were resuscitated under appropriate conditions in BSK-II medium, where apparently all spirochetes showed the typical spiral morphology. This suggests that these atypical forms may be viable Borrelia forms. Despite that, under the present experimental conditions, we cannot exclude whether such growth may represent propagation of some residual spiral forms, previous observations (7, 8) showing that cystic forms of spirochetes can revert into vegetative form suggest that at least part of the pleomorphic forms observed may revert into vegetative form. That *Borrelia burgdorferi *was successfully cultivated from brains of the three patients with Lyme neuroborreliosis in BSK-II medium where pleomorphic and cystic forms were observed in the brain [3,51,52,72,73] suggests that at least part of the persisting spirochetes are viable. The typical spiral form of these cultivated *Borrelia burgdorferi *spirochetes in addition to the present Figure 1 E-H was previously illustrated (52, 73). Whether the polymorphic forms observed in the brains of these three patients may correspond to living, degenerating or "dormant" spirochetes remains to be determined. Further studies will be necessary to analyze whether individual Borrelia cysts taken from the affected brain may revert into vegetative form.

That the ADB1 strain invades neurons and astrocytes *in vitro *indicates that these surviving cultivatable spirochetes are still virulent.

The accumulation of immunocompetent HLA-DR positive microglia and reactive astrocytes in the cerebral cortex of these patients clearly indicates the presence of chronic inflammation as previously suggested [51]. Indeed, *Treponema pallidum, Borrelia burgdorferi *and their lipoproteins evoke cytokine responses in cells of the monocytes/macrophage lineage as well as initiate complement activation. The response elicited by the major membrane lipoproteins of *Treponema pallidum *and *Borrelia burgdorferi *was analogous to that observed with whole bacteria. The vegetative and cystic forms including the vesicular blubs and free vesicular structures of *Borrelia burgdorferi *all contain the biologically active spirochetal surface proteins indicating that they all elicit inflammatory responses including complement activation [reviewed in [73]].

The clinical and the pathological hallmarks of Alzheimer's disease, including beta-amyloid deposition are also present in the atrophic form of general paresis and in tertiary Lyme neuroborreliosis [3,63,72-74]. The facts that *Borrelia burgdorferi *spirochetes were cultivated from the brains of these patients, that Borrelia antigens and genes were co-localized with beta-amyloid deposits in cortical spirochetal colonies, and that the serology of these patients was positive for *Borrelia burgdorferi *are evidences that the present cases correspond to the atrophic parenchymatous form of late Lyme neuroborreliosis.

## Conclusion

Dark field microscopy, histochemical, immunohistochemical and atomic force microscopy (AFM) analyses revealed that pleomorphic and cystic *Borrelia *forms were induced by the various unfavorable conditions that were employed. Extra- and intracellular atypical and cystic forms were observed in neuronal and astrocytic cultures following 1 week of exposure to *Borrelia burgdorferi *(B31 and ADB1). Identical extra- and intracellular atypical and cystic Borrelia forms were also observed in the brains of all three patients with Lyme neuroborreliosis, which were also similar to the atypical forms of *Treponema pallidum *in the brains of patients with general paresis. Astrocytes infected with *Borrelia burgdorferi *exhibited nuclear fragmentation.

Our results suggest that pleomorphic forms, including cystic forms of *Borrelia burgdorferi *may persist in the brain and may explain the long latent stage and persisting infection in Lyme neuroborreliosis. The identification of these extra- or intracellular atypical, cystic and granular forms of *Borrelia burgdorferi *is essential for the histopathological diagnosis of Lyme disease as they may indicate chronic Borrelia infection, even in cases where the typical coiled spirochetes are apparently absent. In analogy to *Treponema pallidum, Borrelia burgdorferi *can persist in the brain in Lyme neuroborreliosis and may initiate and sustain chronic inflammation and tissue damage.

## Competing interests

The authors declare that they have no competing interests.

## Authors' contributions

JM contributed to the direction of the investigation, data interpretation and to the writing of the manuscript. SK contributed to the AFM analysis, ADZ contributed in experiments of neuronal cell cultures, SMC contributed in the analysis of syphilitic brains and in organizing the interaction of several laboratories, as well as in data interpretation, SY contributed to the immunohistochemical analysis, PLMG contributed to the writing of the manuscript and data interpretation. All authors read and approved the final manuscript.
